# Correlation between physical activity levels and the risk of cognitive impairment in Chinese older adults

**DOI:** 10.3389/fnagi.2025.1519494

**Published:** 2025-05-26

**Authors:** Feng-Wei Dong, Dong-Hui Wang, Yu-Jie Chang, Li-Xu Tang

**Affiliations:** ^1^Martial Arts Academy, Wuhan Sports University, Wuhan, Hubei, China; ^2^Department of Physical Education, Yuncheng University, Yuncheng, Shanxi, China

**Keywords:** elderly population, physical activity, cognitive impairment, types of activities, health protection

## Abstract

**Objective:**

To analyze the correlation between the level of physical activity and the risk of cognitive impairment in Chinese older adults aged 60 years and above, and to provide correlational evidence for the development of targeted strategies to prevent cognitive impairment.

**Methods:**

This study used five rounds of longitudinal data from the China Health and Retirement Longitudinal Study (CHARLS) conducted between 2011 and 2020, which included 3,583 older adults aged 60 years and above. Multiple regression models were employed to evaluate the association between varying intensities of physical activity (low-intensity, moderate-intensity, and high-intensity) and the risk of developing cognitive impairment (HR).

**Results:**

In models that were not adjusted for any variables, the risk of cognitive impairment was reduced by 25.3% in the moderate-intensity physical activity group compared to the low-intensity group (HR = 0.747, 95%CI: 0.617–0.903), and by 11.0% in the high-intensity group (HR = 0.890, 95%CI: 0.798–0.992). In the model that fully controlled for all confounding variables, the risk ratio was further reduced to 30.7% (HR = 0.693, 95%CI: 0.571–0.841) in the moderate-intensity physical activity group and 9.7% (HR = 0.903, 95% CI: 0.809–1.007) in the high-intensity group. Dose–response analysis revealed an optimal strength of association between moderate-intensity physical activity (approximately 2,800 MET-minutes per week) and cognitive health.

**Conclusion:**

Moderate-intensity physical activity can significantly reduce the risk of cognitive impairment among older adults in China. It is recommended that health management and cognitive impairment prevention strategies for this population incorporate moderate-intensity physical activity.

## 1 Introduction

With the accelerated aging of the global population, the incidence of cognitive disorders, particularly dementia, has been increasing year after year, placing immense pressure on public health systems and the socio-economic landscape. According to the World Health Organization (WHO), the global population of individuals aged 60 years and older is projected to exceed 2 billion by 2050 (World Health Organization, 2021). In China, which has the largest elderly population in the world, the burden of cognitive impairment has been especially severe. Epidemiological statistics indicate that by the end of 2024, China’s population aged 60 and above had reached 290 million, with approximately 15.90 million cases of dementia (National Health Commission of the People's Republic of China, 2021). This results in a prevalence rate of 6%, the highest in the world. It is anticipated that the number of dementia patients will surpass 40 million by 2040, accounting for about one-quarter of the global population (The State Council, 2022).

Cognitive impairment encompasses two categories: Mild Cognitive Impairment (MCI) and dementia. MCI represents a transitional stage of cognitive decline, characterized by impairments in memory or other cognitive functions, while the ability to perform daily living tasks remains largely intact, and the diagnostic criteria for dementia have not yet been fulfilled. Research indicates that over half of MCI patients will progress to dementia within 5 years (Alzheimer's Association, 2023; Zeisel et al., 2020). Consequently, early intervention for MCI holds significant public health importance and can effectively delay or prevent the onset of dementia (Aarsland et al., 2021). The etiology of cognitive impairment is multifaceted, involving a range of biological, psychological, and socio-environmental factors. In recent years, an increasing number of studies have focused on the relationship between lifestyle factors and cognitive function, with physical activity receiving significant attention as a modifiable lifestyle factor (Dominguez et al., 2021; Sewell et al., 2021). In its initial guidelines for reducing the risk of dementia and cognitive decline, the World Health Organization (WHO) strongly recommended physical activity as a crucial intervention for the prevention of cognitive impairment. International longitudinal cohort studies have provided substantial evidence for the association between physical activity and cognitive impairment. However, their findings are significantly influenced by various regional cultures, measurement methods, and population characteristics (Sewell et al., 2024; Bloomberg et al., 2023). Additionally, there is considerable heterogeneity in the protective effects of physical activity. The Survey of Health, Ageing and Retirement in Europe (SHARE) is a prospective cohort study encompassing 11 countries. It employs a multistage stratified sampling method to evaluate cognitive functioning and physical activity levels through standardized questionnaires administered every 2 years, with an average follow-up period of 12 years (Marques et al., 2021). The research team utilized a mixed-effects model to incorporate various policy variables, including healthcare expenditure, pension coverage, and community care accessibility. This analysis aimed to examine the policy heterogeneity between countries, contrasting universal healthcare in Northern Europe with the predominance of family healthcare in Southern Europe. The study seeks to elucidate the moderating role of the social welfare system on the protective effects of physical activity (Mattle et al., 2022). The use of biomarkers to validate the associations between physical activity and cognition, along with the integration of serum BDNF level testing, revealed that the moderate-intensity activity group had higher BDNF concentrations than the control group. This finding supports the mediating role of neurotrophic mechanisms (O’Donovan et al., 2023). The study’s main findings and strengths include the effects of cultural diversity and long-term follow-up (Sugita et al., 2021). It identifies differences in the impact of family-centered multigenerational activity patterns in Southern Europe compared to structured exercise in Northern Europe, such as family gardening in Spain and community fitness classes in Sweden. These results confirm that social support networks enhance the neuroprotective efficacy of physical activity (Vara-García et al., 2023; Gyrling et al., 2021). Twelve years of follow-up demonstrated that sustained moderate-intensity activity (150–300 min per week) significantly reduces the risk of dementia. However, reliance on self-reported physical activity may overestimate the actual metabolic load. Additionally, the failure to differentiate between occupational (e.g., agricultural labor) and recreational types of activity may result in a biased estimation of the effects of high-intensity activity. The U.S. Health and Retirement Study (HRS), the most representative aging cohort in the nation, has enrolled over 20,000 participants aged 50 years and older since its inception in 1992. The study employs multistage stratified probability sampling and a biennial mixed survey model. Research findings indicate that high levels of physical activity (greater than 4,500 MET-min/week) are associated with a significant reduction in the risk of cognitive impairment; however, the dose–response curve reveals a plateau effect (Manly et al., 2022). The study design includes several notable features: the introduction of accelerometers to objectively measure physical activity and the conversion of raw data into MET values using Freedson’s formula for older adults, which allows for precise quantification of activity intensity (Langa et al., 2020). Using inverse probability weighting to adjust for differences in healthcare resource accessibility through Medicare data linkage, high activity levels (greater than 4,500 MET-min/week) were found to be associated with a reduced risk of cognitive impairment in a highly educated white population (Domingue et al., 2023). The development of the HRS-EXCODE system, which encodes a wide range of activity types, confirmed that the neuroprotective effects of recreational exercise were significantly greater than those of occupational activities (Buzdagli et al., 2024; Bone et al., 2022). Key findings and strengths include the use of accelerometer measurement techniques and specific coding systems that distinguish between occupational labor and recreational exercise, as well as the utilization of Medicare data to analyze the moderating effects of healthcare resource allocation on activity outcomes. However, the racial homogeneity of the sample limits the global generalizability of the results, and there was insufficient control for comorbidities, with no full adjustment for baseline depressive symptoms. The Japan Gerontological Evaluation Study (JAGES) in Asia is the largest geriatric health tracking cohort in Japan, encompassing all 47 prefectures. This cohort is monitored every 3 years using a prospective design. JAGES features a unique cultural profile and has developed a culturally appropriate physical activity scale (Masuko et al., 2025). This scale categorizes gardening and light housework as moderate-intensity activities (MET = 3.5), which is lower than the Western scale (MET = 4.0). This adjustment aligns more closely with the predominantly low-intensity labor lifestyle of older adults in Japan, suggesting that the socio-cultural context of different types of physical activity may influence their health effects (Sugita et al., 2021). By linking to the nursing care insurance database, a community nursing care density index was created to quantify the moderating effects of nursing care facilities and insurance reimbursement coverage on physical activity at both municipal and village levels (Sato et al., 2021). The strengths of this study include the segmentation of localized activity types, which accurately captures the perceived conservation value of culturally distinctive activities in East Asia (e.g., farming, traditional rituals). Additionally, it reveals the amplifying effect of accessibility to intermediary services on the benefits of physical activity, as measured by the Community Intermediary Service Density Index (CISDI). This index provides a focal point for targeted policy interventions (Nofuji et al., 2023). However, the study has limitations, such as the failure to differentiate between occupational labor and leisure activities, which may result in an overestimation of the effects of high-intensity activities. Furthermore, insufficient control of chronic disease comorbidities may introduce confounding effects on the observed impacts of physical activity. All of the studies mentioned above are prospective longitudinal cohort studies, which are less focused on exploring the correlation between physical activity and cognitive dysfunction in older Chinese adults. Although several investigations have been conducted by Chinese scholars, most of these are cross-sectional studies that exhibit limitations in study design and methodology. For instance, Zhao et al. (2025) demonstrated a negative association between physical activity and the risk of cognitive impairment in a cross-sectional analysis based on a community sample (Zhao et al., 2025). Additionally, Li et al.'s (2020) 3-year follow-up study may have been biased in its risk estimation due to the failure to control for chronic disease comorbidities at baseline (Li et al., 2020). Therefore, there is an urgent need to analyze the dose–response relationship using nationally representative longitudinal data, combined with causal inference methods, and to test culture-specific hypotheses within a global evidence framework.

We utilized five rounds of longitudinal data from the China Health and Retirement Longitudinal Study (CHARLS) conducted between 2011 and 2020, selecting 3,583 older adults aged 60 years and above as study subjects. We employed multiple regression models to systematically analyze the correlation between varying levels of physical activity intensity (low-intensity, moderate-intensity, and high-intensity) and the risk of developing cognitive impairment (HR), while controlling for various confounding factors through the propensity score matching (PSM) method. Compared to existing studies, this research presents several innovations: (1) the use of large-scale longitudinal data enhances the representativeness of the findings and improves the understanding of the relationship between physical activity levels and the risk of cognitive dysfunction; (2) the categorization of physical activity intensity clarifies the differential effects of varying intensities on the risk of cognitive impairment; and (3) multiple physiological and psychosocial factors are considered collectively, providing a more comprehensive analytical framework. This study establishes a correlational foundation for developing effective strategies to prevent cognitive impairment among older Chinese adults. It aims to enhance cognitive health in this population and contribute to the sustainable development of an aging society.

## 2 Methods

### 2.1 Data sources and study design

The data utilized in this study were derived from the China Health and Retirement Longitudinal Study (CHARLS). CHARLS is conducted by the National Development Research Institute (NDI) at Peking University and has been carried out every 2–3 years since 2011 as a longitudinal tracking survey of middle-aged and elderly individuals aged 45 and older nationwide, ensuring a high degree of national representativeness. The baseline survey encompasses 450 villages across 150 counties in 28 provinces (including autonomous regions and municipalities directly under the central government) and gathers detailed individual and household information from approximately 17,000 respondents. This information includes physical activity levels, cognitive function, self-rated health status, and demographic variables such as gender, age, marital status, and years of education (Zhou et al., 2022). This comprehensive dataset provides a robust foundation for the present study, which aims to explore the correlation between physical activity and the risk of cognitive impairment in older adults. All data collection processes received approval from the Biomedical Ethics Review Board of Peking University (Approval No. IRB00001052-11015) and strictly adhered to the ethical principles outlined in the Declaration of Helsinki. All participants gave informed consent.

**Figure 1:**
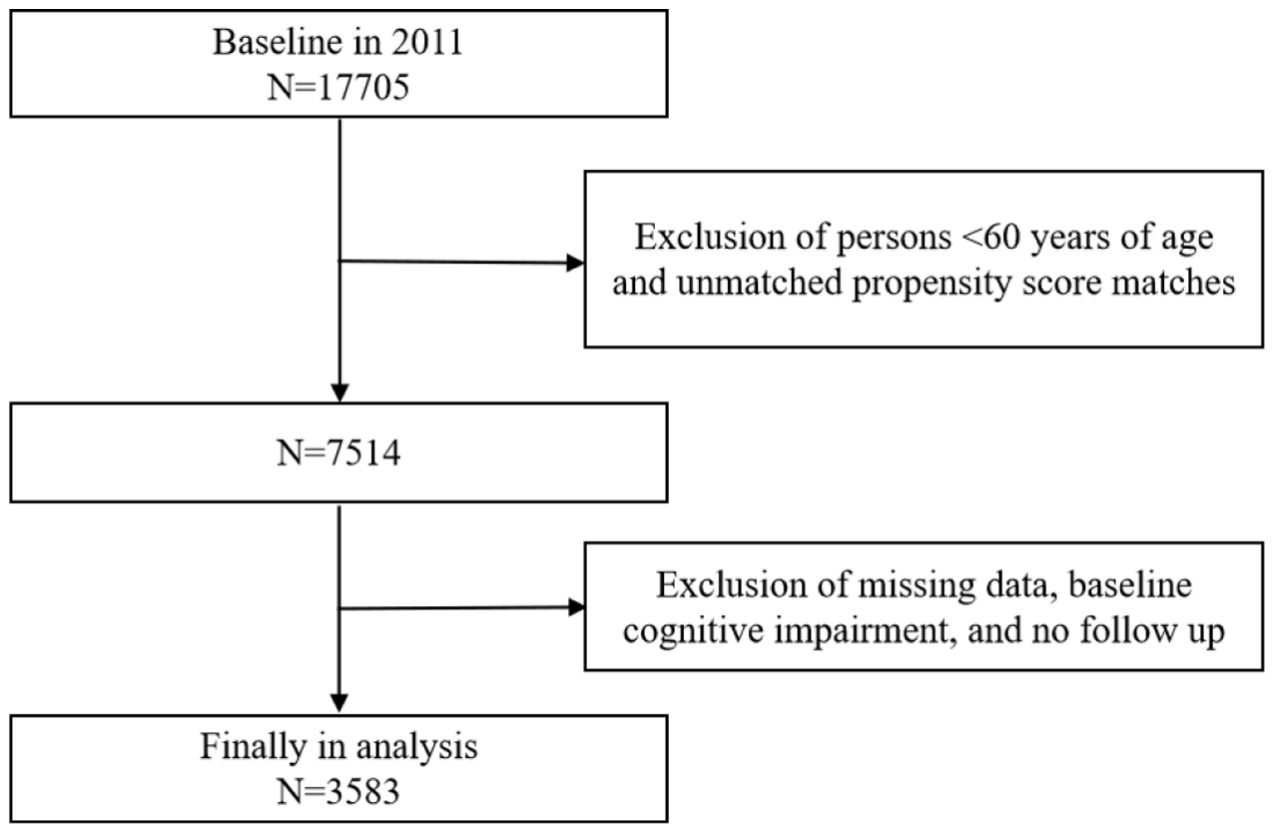
Flowchart of sample selection.

The data included in this study encompassed five rounds of follow-up surveys conducted from 2011 to 2020.The specific sample screening process was as follows: (1) Based on the 17,705 respondents from the 2011 baseline survey, 9,945 individuals younger than 60 years were excluded to ensure that the study population consisted solely of those aged 60 and above. To minimize sample selection bias, the Propensity score matching (PSM) method was employed to match the treatment groups, resulting in the exclusion of 246 respondents who could not be successfully matched during this process. (2) Exclude 2,268 respondents with missing data on the physical activity assessment, 1,242 respondents with missing data on the cognitive function rating, and 114 respondents with pre-existing affective or psychiatric disorders, or memory-related disorders at baseline. Additionally, 307 respondents who did not participate in any subsequent follow-up rounds were excluded. Ultimately, 3,583 older adults who met all inclusion criteria were identified for analysis in this study. The sample was screened using rigorous inclusion and exclusion criteria to ensure that none of the study participants were cognitively impaired at baseline and to eliminate participants with generalized cognitive impairment. The sample screening process is detailed in Figure 1.

### 2.2 Measurement of variables and statistical analysis

#### 2.2.1 Measurement of physical activity level

In this study, data collected from the ‘Lifestyle’ and ‘Health behavior’ sections of the CHARLS questionnaire were utilized to assess the physical activity levels of the respondents. First, respondents were asked to review and report the frequency and duration of their engagement in various types of physical activities over the past week. The frequency of physical activity was categorized into four levels: 0 days per week, 1–2 days per week, 3–5 days per week, and 6–7 days per week (Wang et al., 2023). According to the criteria of the International Physical Activity Questionnaire-Short Form (IPAQ-SF), the duration of daily physical activity was classified into five categories: 0 min, 10–29 min, 30–119 min, 120–239 min, and 240 min or more. The median value of each category was used for subsequent calculations. Secondly, different types of physical activities were assigned corresponding metabolic equivalent (MET) values to quantitatively measure their intensity. The specific values were designated as follows: the MET value for high-intensity physical activities, such as climbing, running, and farming, was set at 8.0; the MET value for moderate-intensity activities, including brisk walking and tai chi, was assigned a value of 4.0; and the MET value for low-intensity activities, such as walking, was established at 3.3. The list of physical activities, along with the methodology for assigning and calculating MET values, is detailed in the Supplementary material. The total MET minutes of physical activity per week were calculated based on the respondents’ reports regarding the frequency and duration of each activity (Herrmann et al., 2024; Kuo et al., 2022). Finally, Based on the total MET minutes per week, the physical activity levels of the respondents were further categorized into: high-intensity physical activity (≥3,000 METs/week), moderate-intensity physical activity (600–3,000 METs/week), and low-intensity physical activity (≤600METs/week).The specific number of participants included 1,245 in the low-intensity physical activity group, 1,732 in the moderate-intensity physical activity group, and 606 in the high-intensity physical activity group.

#### 2.2.2 Measurement of cognitive impairment

Cognitive impairment, the primary outcome variable of this study, was assessed using the Chinese version of the Mini-Mental State Examination (MMSE) as revised by Zhang (1998). This tool was selected to evaluate the respondents’ cognitive levels by calculating their overall scores across four dimensions: date cognition, recall ability, numeracy ability, and drawing ability. The scoring for each dimension is as follows: date cognition (0–5), recall ability (0–20), numeracy ability (0–5), and drawing ability (0–1). The total score ranges from 0 to 31, with higher scores indicating better cognitive ability. Additionally, to accurately identify the presence of cognitive impairment, study participants were categorized based on their educational levels (Fostein et al., 1975). Based on the MMSE rating criteria, the following three conditions were identified as indicators of cognitive impairment among the respondents; otherwise, it was determined that there was no cognitive impairment: (1) Illiteracy with a cognitive score of less than 17; (2) Primary education with a cognitive score of less than 20; (3) Secondary education or higher with a cognitive score of less than 24.

#### 2.2.3 Selection and measurement of covariates

When examining the correlation between physical activity levels and the risk of cognitive impairment, potential confounders can significantly impact the results. To ensure the internal validity of the analysis, this paper references the related studies by He Nanfu and Zhao Ming. The covariates were screened based on basic individual information and chronic disease levels, considering the accessibility and completeness of the variables. The covariates related to basic individual information included age, gender, years of education, marital status, place of residence, smoking status, alcohol consumption, and sleep duration. Chronic disease covariates included the presence of hypertension, lung disease, liver disease, heart problems, stroke, kidney disease, asthma, and cancer. These variables encompass a broad spectrum of socio-demographic, lifestyle, and health status factors, ensuring an accurate representation of the relationship between physical activity levels and the risk of cognitive impairment. The specific selection of variables is presented in Table 1.

**Table 1:** Selection of variables.

Types of variables	Variable name	Variable symbol	Assignment description
Explained variables	Cognitive impairment	outcome	The presence of cognitive impairment is assigned a value of 1; otherwise assigned a value of 0.
Explanatory variables	Physical activity	METs	Metabolic equivalent of physical activity, reduced by a factor of 1,000
Covariates	Age	age	The respondent’s actual age
	Gender	gender	Assign a value of 1 to males and a value of 0 to females.
	Years of education	eduyear	Translating education level: no schooling, primary school, junior high school, high school, junior college, college, bachelor’s degree, master’s degree, and doctoral degree into years of education: 0, 6, 9, 12, 13, 15, 16, 19, and 22 years.
	Marital status	marry	Married is assigned a value of 1, others are assigned a value of 0.
	Place of residence	urban	Urban is assigned a value of 1, rural is assigned a value of 0
	Smoking history	smoke	Yes is assigned 1, No is assigned 0
	Drinking history	drink	Yes is assigned 1, No is assigned 0
	Sleeping hours	sleept	Less than 6 h per day (short duration) is assigned a value of 1; 6 to 8 h per day (normal duration) is assigned a value of 2; and more than 8 h per day (long duration) is assigned a value of 3.
	History of hypertension	hypertension	Yes is assigned 1, No is assigned 0
	History of lung disease	lungdis	Yes is assigned 1, No is assigned 0
	History of liver disease	liverdis	Yes is assigned 1, No is assigned 0
	History of heart disease	heartdis	Yes is assigned 1, No is assigned 0
	History of stroke	stroke	Yes is assigned 1, No is assigned 0
	History of kidney disease	kidneydis	Yes is assigned 1, No is assigned 0
	History of asthma	asthma	Yes is assigned 1, No is assigned 0
	History of cancer	cancer	Yes is assigned 1, No is assigned 0

#### 2.2.4 Propensity score matching method

To effectively control for potential confounders and reduce sample selection bias, this study employed the Propensity Score Matching (PSM) method to align the experimental group (characterized by varying levels of physical activity) with the control group. The one-to-one Nearest Neighbor Matching (NNM) technique was utilized, with a caliper set at 0.2 times the standard deviation, and matching was conducted without replacement. The results of the balance test, conducted before and after matching, are presented in Table 2. The findings indicate that the standardized mean differences for all control variables after matching were reduced to less than 0.1, and the absolute values of the T-statistics were significantly diminished, with most *p*-values being insignificant (*p* > 0.05). This suggests that the experimental group was well-balanced with the control group across all control variables following the matching process. Furthermore, the substantial reduction in the deviation rate further corroborates the effectiveness of the matching procedure.

**Table 2:** Balance test.

Variable name	Matching situation	Mean value of experimental group	Control group mean value	Deviation rate	Deviation reduction rate	*t*-value	*p*-value
age	Before matching	66.385	64.884	31	96.5	9.64	0
	After matching	66.34	66.287	1.1		0.31	0.753
gender	Before matching	0.62144	0.55545	13.4	77.9	4.15	0
	After matching	0.62094	0.60635	3		0.9	0.371
eduyear	Before matching	6.1725	4.6811	33.1	95.4	10.27	0
	After matching	6.1596	6.2278	−1.5		−0.44	0.657
marry	Before matching	0.8364	0.88911	−15.4	88.4	−4.77	0
	After matching	0.83875	0.84484	−1.8		−0.5	0.618
urban	Before matching	0.48409	0.46565	3.7	98.7	1.14	0.254
	After matching	0.48432	0.48409	0		0.01	0.989
smoke	Before matching	0.49749	0.44603	10.3	81.8	3.19	0.001
	After matching	0.4972	0.48784	1.9		0.56	0.576
drink	Before matching	0.38638	0.34789	8	59.6	2.47	0.014
	After matching	0.38522	0.36965	3.2		0.96	0.337
sleept	Before matching	1.9324	1.947	−2.1	−5.1	−0.64	0.521
	After matching	1.9339	1.9186	2.2		0.67	0.505
hypertension	Before matching	0.28308	0.27331	2.2	31.1	0.67	0.501
	After matching	0.28331	0.27658	1.5		0.45	0.654
lungdis	Before matching	0.12172	0.11973	0.6	−617.2	0.19	0.85
	After matching	0.12206	0.10776	4.4		1.34	0.18
liverdis	Before matching	0.04411	0.05103	−3.3	91.8	−1	0.317
	After matching	0.04423	0.0448	−0.3		−0.08	0.934
heartdis	Before matching	0.1435	0.14279	0.2	−2813.4	0.06	0.95
	After matching	0.1439	0.12326	5.9		1.81	0.07
stroke	Before matching	0.01954	0.02944	−6.4	94.4	−1.97	0.049
	After matching	0.0196	0.01904	0.4		0.12	0.904
kidneydis	Before matching	0.06868	0.05839	4.2	80.6	1.31	0.192
	After matching	0.06831	0.07031	−0.8		−0.23	0.814
asthma	Before matching	0.04467	0.03827	3.2	64.6	0.99	0.321
	After matching	0.04479	0.04253	1.1		0.33	0.741
cancer	Before matching	0.01117	0.01178	−0.6	−61	−0.18	0.86
	After matching	0.0112	0.01218	−0.9		−0.27	0.785

### 2.3 Statistical methods

This study used Stata 16.0 software for statistical analysis. On the one hand, the basic characterization of the samples were analyzed using descriptive statistics. Continuous variables were expressed as mean ± standard deviation (Mean ± SD), while categorical variables were presented as frequencies and percentages. To compare the differences between the cognitively impaired group and the non-cognitively impaired group, an independent samples t-test was employed for continuous variables, and a chi-square test was used for categorical variables. Additionally, a Cox proportional hazards regression model was applied to assess the association between different levels of physical activity and the risk of developing cognitive impairment. To explore the dose–response relationship between physical activity and the risk of cognitive impairment, nonlinear analysis were conducted using the restricted cubic splines (RCS) model. The RCS model was utilized to capture the nonlinear effect of physical activity on the risk of cognitive impairment by setting knots at key tertiles to illustrate its dose–response curve. On the other hand, to control for potential confounders, a total of five models were established in the Cox regression analysis, as follows: Model 1 did not include any covariates; Model 2 included basic individual information covariates (age, gender, years of education, marital status); Model 3 incorporated all basic individual information covariates (including place of residence, smoking history, alcohol consumption history, and hours of sleep); Model 4 was based on Model 3 and included additional chronic disease covariates (hypertension, history of lung disease, history of liver disease, and history of heart disease); and Model 5 included all individual basic information and chronic disease covariates (including stroke, kidney disease, asthma, and cancer). All models underwent Schoenfeld residual tests to assess the proportional hazards assumption. The significance level was established at *p* < 0.05.

## 3 Results

### 3.1 Basic characterization of the sample

Table 3 illustrates the distribution of basic characterization among the total sample, the non-cognitively impaired group, and the cognitively impaired group. The overall study sample comprised 3,583 older adults without cognitive impairment at baseline, with a cumulative follow-up of 20,000 person-years and a mean follow-up duration of 5.6 years. During the follow-up period, a total of 1,705 cases of cognitive impairment were identified, resulting in an incidence density of 85 cases per 1,000 person-years. Of these, 1,878 individuals in the non-cognitively impaired group did not develop cognitive impairment during the follow-up period, while 1,705 individuals were classified in the cognitively impaired group.

**Table 3:** Basic characterization analysis.

Major category	Subcategory	Total sample (*n* = 3,583)	Non-cognitively impaired sample (*n* = 1878)	Cognitively impaired sample (*n* = 1705)	χ^2^/T statistic	*p*-value
Age	–	65.559 ± 4.764	64.996 ± 4.312	66.179 ± 5.147	−7.481	0.000
Years of education	–	5.422 ± 4.516	4.862 ± 4.289	6.038 ± 4.678	−7.850	0.000
Gender	Female	1,473(41.11)	820(43.66)	653(38.30)	10.622	0.001
	Male	2,110(58.89)	1,058(56.34)	1,052(61.70)		
Marital status	Others	482(13.45)	216(11.50)	266(15.60)	12.900	0.000
	Married	3,101(86.55)	1,662(88.50)	1,439(84.40)		
Place of residence	Rural	1887(52.67)	1,004(53.46)	883(51.79)	1.002	0.317
	Town	1,696(47.33)	874(46.54)	822(48.21)		
Smoking history	No	1890(52.75)	1,028(54.74)	862(50.56)	6.270	0.012
	Yes	1,693(47.25)	850(45.26)	843(49.44)		
Drinking history	No	2,275(63.49)	1,227(65.34)	1,048(61.47)	5.772	0.016
	Yes	1,308(36.51)	651(34.66)	657(38.53)		
Sleeping hours	Short duration	999(27.88)	496(26.41)	503(29.50)	7.376	0.025
	Normal duration	1824(50.91)	996(53.04)	828(48.56)		
	Long duration	760(21.21)	386(20.55)	374(21.94)		
History of hypertension	No	2,596(72.45)	1,372(73.06)	1,224(71.79)	0.719	0.396
	Yes	987(27.55)	506(26.94)	481(28.21)		
History of lung disease	No	3,162(88.25)	1,663(88.55)	1,499(87.92)	0.346	0.556
	Yes	421(11.75)	215(11.45)	206(12.08)		
History of liver disease	No	3,417(95.37)	1789(95.26)	1,628(95.48)	0.101	0.751
	Yes	166(4.63)	89(4.74)	77(4.52)		
History of heart disease	No	3,072(85.74)	1,618(86.16)	1,454(85.28)	0.562	0.453
	Yes	511(14.26)	260(13.84)	251(14.72)		
History of stroke	No	3,506(97.85)	1835(97.71)	1,671(98.01)	0.371	0.542
	Yes	77(2.15)	43(2.29)	34(1.99)		
History of kidney disease	No	3,357(93.69)	1768(94.14)	1,589(93.20)	1.354	0.245
	Yes	226(6.31)	110(5.86)	116(6.80)		
History of asthma	No	3,435(95.87)	1804(96.06)	1,631(95.66)	0.361	0.548
	Yes	148(4.13)	74(3.94)	74(4.34)		
History of cancer	No	3,543(98.88)	1857(98.88)	1,686(98.89)	0.0001	0.991
	Yes	40(1.12)	21(1.12)	19(1.11)		

*t*-values are used for continuous variables and χ^2^ values are used for categorical variables.

First, an independent samples t-test was conducted to analyze the differences between the non-cognitively impaired group and the cognitively impaired group regarding age and years of education. The results indicated significant differences between the two groups in both age (*t* = −7.481, *p* < 0.001) and years of education (*t* = −7.850, *p* < 0.001). Specifically, the mean age and years of education in the cognitively impaired group were significantly lower than those in the non-cognitively impaired group.

Second, the chi-square test was employed to evaluate the differences between the two groups for categorical variables. The results indicated significant differences in gender (χ^2^ = 10.622, *p* = 0.001), marital status (χ^2^ = 12.900, *p* < 0.001), smoking history (χ^2^ = 6.270, *p* = 0.012), drinking history (χ^2^ = 5.772, *p* = 0.016), and sleep duration (χ^2^ = 7.376, *p* = 0.025) between the cognitively impaired group and the non-cognitively impaired group. Specifically, the cognitively impaired group exhibited a higher proportion of males, a lower proportion of married individuals, a higher proportion of smokers and alcohol consumers, and more respondents reporting either short or long sleep durations.

In comparison, the following factors did not show any significant differences between the two groups: residence (χ^2^ = 1.002, *p* = 0.317), history of hypertension (χ^2^ = 0.719, *p* = 0.396), history of lung disease (χ^2^ = 0.346, *p* = 0.556), history of liver disease (χ^2^ = 0.101, *p* = 0.751), history of heart disease (χ^2^ = 0.562, *p* = 0.453), history of stroke (χ^2^ = 0.371, *p* = 0.542), history of kidney disease (χ^2^ = 1.354, *p* = 0.245), history of asthma (χ^2^ = 0.361, *p* = 0.548), and history of cancer (χ^2^ = 0.0001, *p* = 0.991).

As shown in the results presented in Table 3, the cognitive impairment group exhibited significant differences from the non-cognitive impairment group regarding lifestyle and sociodemographic characteristics, including gender, marital status, smoking history, drinking history, and sleep duration. We conducted subgroup analyses based on demographic characteristics, lifestyle, and health status. Age subgroups indicated that the protective effect was significantly stronger in the 60–69 year age group (HR = 0.62, 95% CI 0.51–0.75) compared to the 70–79 year age group (HR = 0.78, 95% CI 0.64–0.95) and the ≥80 year age group (HR = 0.92, 95% CI 0.77–1.10). This attenuation may be associated with decreased neuroplasticity and a lower metabolic rate. As individuals age, the annual rate of volumetric atrophy in the hippocampus increases, resulting in diminished synaptic plasticity and a reduced ability of physical activity to enhance cognitive reserve (De Felice et al., 2022). Additionally, with advancing age, the basal metabolic rate declines, leading to an increased metabolic load threshold required for physical activity, necessitating higher-intensity exercise to stimulate BDNF secretion (Ross et al., 2023). There was a significant interaction between education level and physical activity. The protective effect was strongest in the low education group (≤6 years) (HR = 0.51, 95% CI 0.44–0.60), followed by the middle education group (7–9 years) (HR = 0.68, 95% CI 0.57–0.81), and weakest in the high education group (≥10 years) (HR = 0.82, 95% CI 0.68–0.98). This aligns with the cognitive reserve theory, which posits that individuals with lower education levels have reduced gray matter density in the cerebral cortex and are more reliant on physical activity to enhance hippocampal-prefrontal functional connectivity (Bliss et al., 2021). This enhancement compensates for their limited cognitive resources. In contrast, individuals in the high education group may be able to lessen their dependence on physical activity by utilizing healthcare resources, such as regular medical checkups and pharmacological interventions. Consequently, the low education group relies more heavily on physical activity as a primary means of maintaining health (Johansson et al., 2022). Furthermore, highly educated individuals facing cognitive challenges are more likely to manage stress through cognitive reappraisal strategies, which may diminish the protective effect of physical activity (Amian et al., 2024). Stratification by marital status revealed that the protective effect was significantly greater in the married group (HR = 0.65, 95% CI 0.56–0.76) compared to the widowed or living alone group (HR = 0.89, 95%CI 0.75–1.05). Possible explanations for this difference include the notion that marital relationships may indirectly enhance cognitive protection by reducing feelings of loneliness, mitigating hippocampal atrophy associated with overactivation of the HPA axis (Sharan and Vellapandian, 2024), and providing spousal support that facilitates physical activity, thereby improving adherence to exercise routines. The stratification of chronic disease comorbidities revealed a significantly greater protective effect in the group without chronic diseases (HR = 0.58, 95% CI 0.50–0.68) compared to the multimorbidity group (HR = 0.81, 95% CI 0.70–0.93). The primary mechanism underlying the 6% decrease in hazard ratio (HR) for each additional chronic disease is the synergistic effect of systemic inflammation and metabolic disorders. Chronic diseases contribute to elevated levels of pro-inflammatory factors, inhibition of brain-derived neurotrophic factor (BDNF) expression, and impaired synaptic plasticity (Sharma et al., 2021). Furthermore, the coexistence of multiple diseases accelerates physiological decline, making it challenging for physical activity to surpass the “threshold effect” on cognitive function (Maltagliati et al., 2025), which may counteract the neuroprotective benefits of physical activity. Regarding lifestyle factors, current smokers exhibited significantly lower effect sizes for physical activity compared to non-smokers. This discrepancy may be attributed to the inhibitory effect of nicotine on synaptic plasticity in the hippocampus. In smokers, the expression of hippocampal nicotinic acetylcholine receptors is downregulated, which inhibits signaling pathways and diminishes the neuroprotective effects of physical activity (Zare et al., 2025). The protective effect of physical activity was more pronounced in moderate drinkers (≤2 times per week) (HR = 0.61, 95% CI 0.50–0.74), but it diminished in heavy drinkers (>3 times per week) (HR = 1.02, 95% CI 0.87–1.20). It suggests that moderate alcohol consumption (up to two times per week) enhances antioxidant capacity and synergizes with physical activity to improve mitochondrial function by upregulating the Nrf2 pathway (Simon and Molina, 2022). However, excessive alcohol intake leads to increased reactive oxygen species (ROS) production, triggering lipid peroxidation and DNA damage, which counteracts the benefits of physical activity (Chen et al., 2023).

### 3.2 Association between physical activity levels and the risk of cognitive impairment

Table 4 presents the estimation results for the hazard ratio (HR) of developing cognitive impairment across five models at varying levels of physical activity (low-intensity, moderate-intensity, and high-intensity). The adjustment of covariates for each model is described in detail in Section 2.3. During the modeling process, we employed the Schoenfeld residual test to verify the proportional hazards assumption for each covariate in the model. The test results indicate that none of the Schoenfeld residuals for any covariate exhibited a significant correlation with time (*p* > 0.05), confirming that there were no violations of the proportional hazards assumption. This finding ensures the applicability and robustness of the Cox model. The following are the key results observed from the model analysis:

**Table 4:** Risk of cognitive impairment at different levels of physical activity.

Model setting	Risk profile	Low-intensity physical activity (*n* = 1,245)	Moderate-intensity physical activity (*n* = 1732)	High-intensity physical activity (*n* = 606)
Model 1	Hazard ratio (HR)	1	0.747	0.890
	95%CI	Reference	0.617–0.903	0.798–0.992
Model 2	Hazard ratio (HR)	1	0.685	0.903
	95%CI	Reference	0.565–0.830	0.809–1.007
Model 3	Hazard ratio (HR)	1	0.690	0.905
	95%CI	Reference	0.569–0.837	0.811–1.009
Model 4	Hazard ratio (HR)	1	0.687	0.904
	95%CI	Reference	0.566–0.833	0.810–1.008
Model 5	Hazard ratio (HR)	1	0.693	0.903
	95%CI	Reference	0.571–0.841	0.809–1.007

HR refers to the hazard ratio, and 95% CI denotes the 95% confidence interval.

Model 1 statistics indicated a 25.3% reduction in the risk of cognitive impairment for the moderate-intensity physical activity group compared to the low-intensity physical activity group (HR = 0.747, 95% CI: 0.617–0.903). Additionally, there was an 11.0% reduction in the risk of cognitive impairment for the high-intensity physical activity group relative to the low-intensity physical activity group (HR = 0.890, 95% CI: 0.798–0.992).

Model 5 statistics indicated a further reduction in the risk of cogenitive impairment to 30.7% in the moderate-intensity physical activity group compared to the low-intensity physical activity group (HR = 0.693, 95% CI: 0.571–0.841). Additionally, there was a 9.7% reduction in the risk of cognitive impairment in the high-intensity physical activity group relative to the low-intensity physical activity group (HR = 0.903, 95% CI: 0.809–1.007); however, this result approached statistical significance (*p* = 0.054).

### 3.3 Analysis of the dose–response relationship

**Figure 2:**
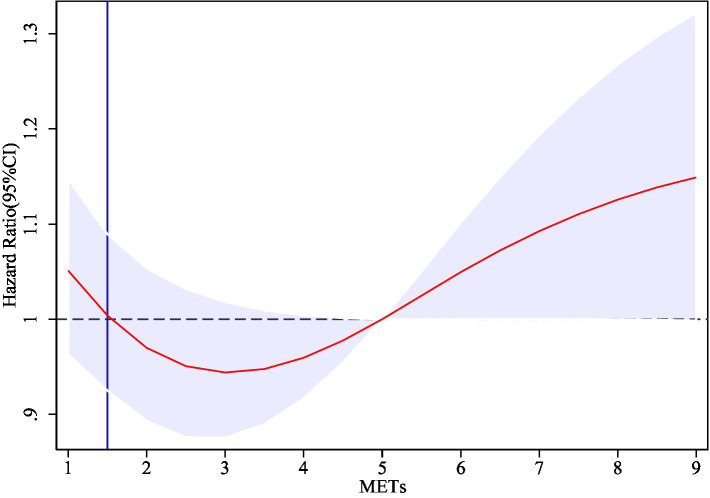
Dose–response curves for physical activity levels and the risk of cognitive impairment.

To thoroughly investigate the dose–response relationship between physical activity levels and the risk of cognitive impairment, this study employed the Restricted Cubic Spline (RCS) model for analysis, utilizing three knots positioned at the 10th, 50th, and 90th percentile levels of physical activity. Figure 2 illustrates the dose–response relationship between physical activity levels (measured in MET-minutes per week) and the risk of cognitive impairment (HR) after adjusting for a number of confounding variables, where 1–9 in the x-axis denote 1,000 METs/week-9000 METs/week, respectively. The solid red line in the figure represents the estimated cognitive impairment risk (HR), and the light blue shaded area is the 95% confidence interval. The results showed a significant nonlinear relationship between physical activity and cognitive impairment risk (HR). Specifically, the risk of cognitive impairment reached its nadir when the level of physical activity was increased to approximately 2,800 METs/week, at which time the HR was approximately 0.94, which was significantly lower than that of the low-intensity physical activity group (HR = 1.00). HR gradually increased as physical activity levels exceeded 3,000 METs per week, suggesting that high-intensity physical activity may no longer confer additional cognitive protective benefits. The findings illustrate the optimal strength of association between moderate-intensity physical activity (approximately 2,800 METs per week) and cognitive health, indicating that moderate-intensity physical activity plays a crucial role in maintaining cognitive health, particularly in preventing cognitive impairment among older adults.

### 3.4 Risk analysis of cognitive impairment by categorical variables

To gain insight into the influence of sociodemographic characteristics and chronic diseases on the risk of cognitive impairment, this paper analyzed the hazard ratios (HR) for cognitive impairment under various categorical variables based on a comprehensive model that includes covariates. Table 5 presents the risk ratios and their 95% confidence intervals for each categorical factor. The specific findings are as follows: the analysis of gender categorization revealed that the probability of cognitive impairment risk increased by 4.4% in male participants compared to female participants; the analysis of marital status indicated that the probability of cognitive impairment risk decreased by 17.9% in married individuals compared to those who were not married. Furthermore, the classification of place of residence showed that the risk of cognitive impairment decreased by 8.1% in urban samples compared to rural samples. Additionally, the results from the classification of chronic diseases indicated that individuals with chronic diseases exhibited a significant increase in the probability of developing cognitive impairment compared to those without chronic diseases.

**Table 5:** Risk of cognitive impairment by classification.

Major category	Subcategory	Hazard ratio (HR)	95%CI
Gender	Female	1	Reference
	Male	1.044	0.913–1.194
Marital status	Others	1	Reference
	Married	0.821	0.716–0.941
Place of residence	Rural	1	Reference
	Town	0.919	0.830–1.018
Smoking history	No	1	Reference
	Yes	1.075	0.951–1.215
Drinking history	No	1	Reference
	Yes	1.039	0.933–1.158
Sleeping hours	Short duration	1	Reference
	Normal duration	0.874	0.781–0.979
	Long duration	0.958	0.836–1.098
History of hypertension	No	1	Reference
	Yes	1.060	0.950–1.183
History of lung disease	No	1	Reference
	Yes	1.023	0.873–1.199
History of heart disease	No	1	Reference
	Yes	1.116	0.921–1.353
History of kidney disease	No	1	Reference
	Yes	1.069	0.832–1.373
History of asthma	No	1	Reference
	Yes	1.044	0.913–1.194

HR refers to the hazard ratio, and 95% CI denotes the 95% confidence interval.

Taken together, the results indicate that factors such as marital status, hours of sleep, and chronic diseases significantly influence the risk of cognitive impairment in the comprehensive model. In contrast, residency and gender, among other variables, exhibited smaller or negligible effects on cognitive impairment when controlling for other factors. These findings underscore the importance of sociodemographic characteristics and lifestyle choices in cognitive health. They suggest that, when developing prevention strategies for cognitive impairment, greater emphasis should be placed on men, unmarried individuals, smokers, drinkers, and older age groups with chronic diseases.

## 4 Discussion

Based on five rounds of longitudinal data from CHARLS between 2011 and 2020, this study systematically explored the correlation between physical activity levels and the risk of developing cognitive impairment in Chinese adults aged 60 and older. The results of the study indicated that moderate-intensity physical activity significantly reduced the risk of cognitive impairment compared to the low-intensity physical activity group. Although the high-intensity physical activity group also demonstrated a reduction in the risk of cognitive impairment, this decrease was significantly smaller than that observed in the moderate-intensity group. Furthermore, dose–response analysis revealed an optimal strength of association between moderate-intensity physical activity and cognitive health.

A comparison of the results from the present study with those of multi-regional studies reveals both commonalities and cultural specificities in the association between physical activity and cognition. (1) The East Asian Cultural Circle: The Moderating Role of Metabolic Equivalent Calibration and Social Activity. Low MET Thresholds and Limitations of Structured Exercise in Japan (Ihira et al., 2022). The dose–response curve from the Japanese Kurita team (2020) indicated a moderate-intensity activity inflection point of 2,500 METs per week, which was significantly lower than the 2,800 METs per week reported in this study (Kurita et al., 2020). This difference may be attributed to several factors: variations in the metabolic equivalent (MET) calibration of different activity types. Typical activities for older adults in Japan, such as gardening and light housework, generally have MET values of ≤3.5. In contrast, activities like farming and lifting or carrying in rural Chinese older adults have MET values of ≥4.0. For instance, 30 min of farming (MET = 4.0) per day in Chinese older adults is equivalent to 45 min of gardening (MET = 3.3) in Japanese older adults. This discrepancy objectively results in a shorter duration of activity required for the Chinese population to achieve similar cognitive protective effects. Additionally, the moderating effect of social activity participation plays a significant role. Chinese older adults tend to have relatively high rates of participation in community-based group activities, such as square dancing and tai chi. The cognitive stimulation associated with these social interactions has not been adequately captured by traditional assessment scales (Kuo et al., 2022). In contrast, the Japanese study did not include such non-exercise activities, leading to an underestimation of the protective effects of moderate-intensity activities. This indicates that a localized activity classification system should be established in East Asia, rather than merely applying Western MET criteria. Korean scholars Yoon et al. (2021) found that the protective effects of physical activity on cognition were significantly greater in women than in men (Yoon et al., 2021), which contrasts with the findings of this study, which reported no gender differences. This discrepancy may arise from two factors: (1) potential bias in measuring non-motor activities, as Korean women are more likely to participate in social activities such as fan dancing, whose cognitive stimulation effects may not be adequately captured by the assessment scale; and (2) the high prevalence of smoking among Chinese men, where nicotine may impair the protective effects of physical activity by inhibiting hippocampal synaptic plasticity (Bao et al., 2024). Therefore, definitions of activity types within cultural contexts should include non-motor cognitive stimuli. (2) European Studies: Interaction Between Policy Interventions and Neural Mechanisms. Northern Europe exhibits a synergistic effect of universal healthcare coverage and structured exercise, as demonstrated by the Swedish cohort (Svensson et al., 2021), which indicates that moderate-intensity activity effectively reduces the risk of dementia. This effect may arise from two key factors: (1) policy-driven healthcare accessibility, where universal healthcare coverage in Nordic countries facilitates cognitive screening and exercise instruction for older adults; and (2) the neural mechanisms associated with structured exercise, where gym training enhances cognition through an increase in hippocampal volume (Nicastri et al., 2022). In contrast, walking and tai chi chuan-based activities in China primarily engage cerebellar-prefrontal pathways to improve executive function (He et al., 2023). Southern Europe also holds potential for cognitive protection through domestic labor. The Spanish JAGES study found that the protective effect of family gardening activities (MET = 3.5) was significantly greater than that of a Nordic fitness program. The underlying mechanisms include: (1) multigenerational social support, where family involvement fosters cross-generational interactions and activates the prefrontal cortex’s default mode network (Jiménez-Roger and Sánchez, 2023); and (2) differences in metabolic load thresholds, as older adults in Southern Europe tend to have higher basal metabolic rates than their counterparts in Northern Europe, necessitating lower-intensity activities to achieve cognitive protection. Furthermore, the higher proportion of individuals with tertiary education in the European high-activity sample compared to China—where access to healthcare resources may obscure the independent effects of physical activity—suggests that various exercise modalities may influence cognitive health through distinct neural mechanisms. (3) Africa: Insights from Studies of Rural High Baseline Activity. Both the Ethiopian (Takele et al., 2024) and Kenyan (Musyimi et al., 2024) studies demonstrated a saturation effect associated with the presence of local subsistence labor. Physical activity was not significantly linked to cognitive impairment, and the underlying mechanisms may include: (1) physiological threshold effects, with MET values of ≥5.0 for local farming and lifting and carrying activities, which far exceed the MET value of 4.0 for the farming activities of elderly rural Chinese. This difference approaches the threshold of maximum capacity of the mitochondrial respiratory chain, potentially leading to an early saturation of cognitive protective effects (Gela et al., 2022); (2) A lack of cognitive stimuli, characterized by predominantly repetitive survival tasks (e.g., fetching water, farming) and an absence of complex movement sequences (e.g., dancing) or social interactions, fails to engage the prefrontal-temporal joint cortex; (3) there is metabolic antagonism related to nutrient-activity. A study conducted in rural Ghana (Awuviry-Newton et al., 2023) revealed that inadequate protein intake, despite extremely high activity levels, led to an imbalance in the tryptophan-kynurenine pathway, resulting in neuroinflammation that counteracts the benefits of exercise. This underscores the necessity for integrated nutrition and activity interventions aimed at enhancing dietary quality while preserving traditional labor practices during urbanization, thereby providing a foundation for tailored intervention strategies in developing countries. (4) The Urban–Rural Dichotomy Model and Exposomic Challenges in Latin America. (1) Cognitive Gains in Urban Leisure Culture. The Brazilian ELSA cohort (Feter et al., 2023) demonstrated that the dose–response inflection point for leisure activities (e.g., samba, soccer) among urban residents was 1800 MET-min/week, significantly lower than that of the current sample (2,800 MET-min/week). The cognitive gains were primarily attributed to the expansion of social networks and, to a lesser extent, the learning of complex movements; (2) Neurotoxicity of Exposure to Rural Environments. The Peruvian cohort (Chino et al., 2022) found that the cognitive protective effect diminished despite an agricultural activity level of 3,000 MET-min/week, which may be linked to neuroinflammation resulting from exposure to organophosphorus pesticides. Studies examining the urban–rural dichotomy, environmental exposure, and methodological differences in Latin America significantly contribute to the global body of evidence. As China’s urbanization rate rises and traditional productive labor declines, the protective effects of recreational sports must be reevaluated (Li et al., 2023). Additionally, air pollution has emerged as a new neurotoxic factor, even as levels of organophosphorus pesticide exposure show a decreasing trend.

The mechanisms through which physical activity influences cognitive impairment are understood via multiple physiological and behavioral pathways. First, regarding physiological mechanisms, moderate-intensity physical activity enhances cerebral blood flow, promotes the expression of brain-derived neurotrophic factor (BDNF), and improves neuroplasticity and synapse formation, thereby delaying neuronal degeneration (De Sá et al., 2024). Additionally, physical activity reduces levels of pro-inflammatory cytokines and mitigates chronic inflammation, both of which are crucial in preventing cognitive decline (Silva et al., 2024). Furthermore, physical activity inhibits *β*-amyloid deposition in the brain and slows the pathological changes associated with Alzheimer’s disease (López-Ortiz et al., 2021). Second, from a metabolic health perspective, physical activity alleviates the burden of chronic diseases such as hypertension and diabetes—significant risk factors for cognitive impairment—by improving glucose metabolism, lowering lipid levels, and managing body weight (Tang et al., 2022). By managing chronic diseases, physical activity indirectly lowers the risk of cognitive impairment. Furthermore, from the standpoint of behavioral habits and social interactions, physical activity often leads to an increase in social engagement and enhances mental health. Participation in team sports or outdoor activities not only fosters social connections but also elevates mood, diminishes negative emotions such as depression and anxiety, and further enhances cognitive function (Zhang and Yue, 2021).

Although this study yielded valuable findings, several limitations should be acknowledged. (1) The assessment of physical activity levels relied on self-reported questionnaires, which may be susceptible to memory and reporting biases. This is particularly true for high-intensity physical activities, where respondents may overreport their activity levels due to inaccurate recall or social desirability effects. Furthermore, older adults with impaired cognitive function may be more vulnerable to these misreporting issues, thereby compromising the accuracy of high-intensity physical activity results. (2) Although propensity score matching (PSM) methods were employed to control for potential confounding variables, unmeasured confounders, such as dietary habits and socio-economic status, may still influence the results. (3) Cognitive impairment was evaluated using the Mini-Mental State Examination (MMSE), which may have limited sensitivity to early mild cognitive impairment and could underestimate the actual prevalence of cognitive impairment. Furthermore, the sample in this study predominantly consisted of older adults from mainland China, necessitating cautious replication of the results in other cultural and geographical contexts.

Based on the findings of this study, the following policy recommendations are proposed: (1) Promote moderate-intensity physical activity. The government and relevant organizations should develop and disseminate physical activity guidelines tailored for the elderly, encouraging moderate-intensity exercises such as brisk walking, tai chi, and square dancing as integral components of daily life. (2) Enhance community support and facility development. Strengthen the construction of community fitness facilities to provide safe and accessible spaces for exercise, and organize regular physical activity programs to encourage active participation among older adults. (3) Implement comprehensive health management. Integrate physical activity into a holistic health management system for the elderly, and create personalized physical activity intervention programs for groups at high risk of cognitive impairment.

In summary, this study emphasized the connection between moderate-intensity physical activity and a reduced risk of cognitive impairment, indicating that engaging in moderate-intensity physical activity may significantly contribute to cognitive health. However, further research is necessary to address the existing limitations and to thoroughly investigate the intricate relationship between physical activity and cognitive health. This will aid in the development of more comprehensive and effective prevention strategies for cognitive impairment.

### Data anonymization and protection

The CHARLS team removed direct identifiers before the data release, retaining only anonymized participant IDs for cross-period tracking and matching. This process ensured that IDs could not be traced back to individuals. All statistical analyses were conducted using group-level data; no individual-level data was stored or shared. Furthermore, the research adhered to the ethical requirements of the Personal Information Protection Act and the Declaration of Helsinki, ensuring that the data was used solely for academic research.

## Data Availability

Publicly available datasets were analyzed in this study. This data can be found here: http://charls.pku.edu.cn/en, The China Health and Retirement Longitudinal Study.
