# Supplementary material for: Correlation between physical activity levels and the risk of cognitive impairment in Chinese older adults
Source: Front Aging Neurosci. 2025 May 26;17:1519494. doi: 10.3389/fnagi.2025.1519494 (PMC12146368; doi:10.3389/fnagi.2025.1519494)
Supplement: Supplementary file 1 [file Table_1.docx]

Supplementary Material

We utilized data collected from the and modules of the China Health and Retirement Longitudinal Study (CHARLS) questionnaire to conduct a comprehensive assessment of the physical activity levels of the interviewees. The specific methods are outlined as follows:

**1.Types of Physical Activity:**

The CHARLS questionnaire encompasses a wide range of physical activity types to accurately capture the respondents' daily activities. Specifically, the following activities are included:

Leisure and Recreational Activities: walking, brisk walking, jogging, hiking, running, tai chi, square dancing, swimming, cycling, aerobic exercises (e.g., dancing, calisthenics)

Household Chores: Sweeping, Mopping, Laundry, Cooking, Gardening

Work-related activities include manual labor, heavy lifting, and agricultural work.

Transportation-related activities include walking, bicycling, and using public transportation.

**2 .Frequency and duration of activities：**

To quantify the intensity of physical activity, various types of activities listed in the questionnaire were assigned corresponding metabolic equivalent (MET) values. The specific format for questioning was as follows:

1. Frequency: average, how many days per week did you engage in [name of activity] during the past week?
2. Duration: “What was the average duration of each session of [name of activity]?”
3. Assignment of Activity Intensity and Metabolic Equivalent (MET):

To quantitatively measure the intensity of physical activity, various activities listed in the questionnaire were assigned corresponding metabolic equivalent (MET) values. Based on the International Physical Activity Questionnaire (IPAQ), MET values were assigned for each minute of the three types of physical activity. The specific criteria for assigning these values are as follows:

High-intensity physical activity: MET value 8.0

Activities that require significant physical exertion include running, fast cycling, climbing, farming, and swimming.

Moderate-intensity physical activity: MET value 4.0

The types of activities include brisk walking, tai chi, square dancing, light gardening, and other intensity exercises.

Low-intensity physical activity: MET value 3.3

Type of Activity: Light physical activities, such as walking, leisurely cycling, and gentle stretching.

The specific formula for calculating physical activity is as follows: (high-intensity duration per day × days per week × 8.0) + (moderate-intensity duration per day × days per week × 4.0) + (low-intensity duration per day × days per week × 3.3).

**3.Calculation of the Average Duration of Physical Activity:**

In our study, physical activity data were collected through self-reports from respondents. Participants were asked to reflect on the various types of physical activities they had engaged in over the past week and to report the frequency and duration of each activity. Specifically, we utilized the International Physical Activity Questionnaire (Short Form) (IPAQ-SF) as a reference standard to categorize the duration of physical activity into five bands: 0 minutes, 10-29 minutes, 30-119 minutes, 120-239 minutes, and ≥240 minutes. The median value of each band was employed for subsequent calculations.

The calculation process is as follows:

1. Respondents reported the frequency of activity by indicating the number of days per week they engaged in a particular activity.
2. The average minutes for each activity were calculated using the median value of the respondents' reported duration brackets as the average number of minutes per activity.
3. The total activity minutes were calculated by multiplying the average duration of each activity by its weekly frequency. The total minutes for all activities were then summed to determine the overall activity time per week.
4. MET Value Calculation: Based on the intensity of various activities, corresponding metabolic equivalent (MET) values are assigned, and the total MET minutes per week are calculated. The formula for calculating MET is: MET value × number of days × number of minutes for each activity. A higher MET value indicates a greater level of physical activity.

**4.Classification of Physical Activity Levels:**

Based on the total MET minutes per week, the physical activity levels of the respondents were further categorized as follows:

High-intensity physical activity (≥3,000 MET minutes per week)

Moderate-intensity physical activity (600-3,000 MET minutes per week)

Low-intensity physical activity (≤600 MET minutes per week)
